# Item Response Theory Analysis of Two Questionnaire Measures of Arthritis-Related Self-Efficacy Beliefs from Community-Based US Samples

**DOI:** 10.1155/2010/416796

**Published:** 2010-06-06

**Authors:** Thelma J. Mielenz, Michael C. Edwards, Leigh F. Callahan

**Affiliations:** ^1^Department of Epidemiology, Mailman School of Public Health, Columbia University, New York, NY 10032, USA; ^2^Department of Allied Health Science, Division of Physical Therapy, School of Medicine, University of North Carolina, Chapel Hill, NC 27599, USA; ^3^Department of Psychology, The Ohio State University, Columbus, OH 43210, USA; ^4^Thurston Arthritis Research Center, University of North Carolina, Chapel Hill, NC 27599, USA; ^5^Departments of Medicine, Orthopedics, and Social Medicine, University of North Carolina, Chapel Hill, NC 27599, USA

## Abstract

Using item response theory (IRT), we examined the Rheumatoid Arthritis Self-efficacy scale (RASE) collected from a People with Arthritis Can Exercise RCT (346 participants) and 2 subscales of the Arthritis Self-efficacy scale (ASE) collected from an Active Living Every Day (ALED) RCT (354 participants) to determine which one better identifies low arthritis self-efficacy in community-based adults with arthritis. The item parameters were estimated in Multilog using the graded response model. The 2 ASE subscales are adequately explained by one factor. There was evidence for 2 locally dependent item pairs; two items from these pairs were removed when we reran the model. The exploratory factor analysis results for RASE showed a multifactor solution which led to a 9-factor solution. In order to perform IRT analysis, one item from each of the 9 subfactors was selected. Both scales were effective at measuring a range of arthritis SE.

## 1. Introduction

The benefits from physical activity to improve arthritis outcomes are well established [1–5]. High self-efficacy (SE) has been shown to be associated with better arthritis health outcomes including adherence to physical activity recommendations [6]. In fact, SE is one of the most important psychosocial determinants of physical activity behavior [7–11]. Bandura's well-known definition of SE is based on social cognitive theory and “focuses on the individual's personal confidence beliefs about his or her capacity to undertake behavior or behaviors that may lead to desired outcomes, such as health” [12]. SE is a task-specific or behavior-specific construct meaning that to increase physical activity, then you only need to focus on SE for physical activity [13, 14]. 

Recent literature suggests the importance of evaluating both SE for a specific task and SE for disease self-care [6, 12]. More specifically, Marks et al. suggested that to be effective, interventions should focus not only on increasing SE for a specific task (e.g., physical activity) but also on enhancing arthritis SE (i.e., disease self-care) [6, 12]. This approach is supported by Kovar et al.'s intervention study evaluating a walking program in patients with knee osteoarthritis. They found that enhancing both physical activity SE and SE for arthritis self-care led to improvements in function without an increase in symptoms [15]. 

Because SE is modifiable, there is increasing interest in interventions. If effective interventions are to be designed to increase SE for arthritis self-management, then accurate measurement of SE is crucial. An on-going challenge has been in identifying people with low SE for disease self-management in sample populations of persons with chronic diseases like arthritis [6, 12]. To assess this precision of SE measurement, we examined two SE for arthritis scales using item response theory (IRT) in participants from two community-based randomized controlled trials (RCTs) on physical activity in adults with arthritis.

IRT represents “a diverse family of models designed to represent the relation between an individual's item response and underlying latent trait” [16]. IRT has several notable benefits. First, in the context of health outcomes and disability, IRT models allow for the differential weighting of items in terms of their severity. IRT also provides item and test information functions. Information functions describe not only how much information is provided by a given item or test, but also where that information is provided. This knowledge can play a crucial role when choosing a scale for a particular purpose. One scale may measure low levels of SE very well but fail to adequately assess higher levels. We hypothesized that the two SE scales studied here will possess different measurement characteristics. These different measurement characteristics will provide guidance in determining which measure is preferred depending on the situation with the overall goal of increasing the precision of SE measurement.

## 2. Materials and Methods

### 2.1. Sample

The first RCT compares outcomes of People with Arthritis Can Exercise (PACE). Detailed methods for the PACE RCT are outlined by Callahan et al. in the main paper [17]. The PACE project team worked in conjunction with the NC Arthritis Program and with community facilities throughout the state including senior centers, assisted living communities, community centers, churches, and wellness centers to recruit participants. The project conducted classes and assessments at 18 sites in counties throughout North Carolina. Class enrollment at the sites ranged from 6 to 34 participants, with a total of 346 participants recruited. The participants had to be exercising <3 times a week for <20 minutes at a time to enroll. The baseline assessments were conducted from August 2003 to November 2003. The demographics included a mean age of 70, 90% female, 75% Caucasian, and 60% had more than a high school degree. Both the baseline and eight-week follow-up assessments involved administering self-report measures on symptoms, function (including physical performance tests), physical activity, and psychosocial outcomes. At the end of the 8-week intervention, study participants in the intent-to-treat analysis showed decreased pain and fatigue and increased arthritis SE [17]. 

Active Living Every Day (ALED) is a 20-week lifestyle program designed to teach behavioral skills to become and stay physically active [18, 19]. The goal of the second RCT was to evaluate ALED compared to a delayed control in individuals with arthritis. The ALED instructors were recruited with the help of the North Carolina Area Agencies on Aging. The instructors were trained in Chapel Hill, NC in December 2003 by one of the original program developers from the Cooper Institute. Three-hundred and fifty-four sedentary (exercising <3 times a week) participants enrolled from 17 urban and rural sites recruited in a similar manner as PACE above. The demographics for this study population include a mean age of 69 years, approximately 80% female, 75% Caucasian, and >50% had more than a high school education. Self-report assessments are on function (including physical performance), symptoms, physical activity, and psychosocial outcomes at baseline and 20-weeks. Two-level (site 2nd level) regression models were used to determine adjusted mean outcome values for the intervention and control groups at 20 weeks. In the intent-to-treat analyses, the intervention group showed improvement over the control group for all outcomes and significant changes for several outcomes including gait speed, 2-minute step, and scores on the Community Healthy Activities Model Program for Seniors (CHAMPS) physical activity scale [18]. 

### 2.2. Measures

The 28-item Rheumatoid Arthritis SE scale (RASE) was completed by PACE participants at baseline and the 8-week follow-up; this study uses the baseline data. The RASE scale measures confidence in one's ability to perform specific self-management behaviors for individuals with all forms of arthritis even though it was initially developed for individuals with rheumatoid arthritis [20, 21]. The scale is self-administered and takes approximately ten minutes to complete. Scores from the RASE are created by summing the 28 items with a five-point Likert response pattern, yielding a possible range of 28 to 140 points. Higher scores indicate higher SE for arthritis self-management [20, 21]. The RASE has demonstrated sensitivity to change following a self-management education program (+5.2, SD 15.5) [20]. The baseline RASE score in the PACE study was 105.05, SD 12.66. 

The 5-item Pain (PSE) and the 6-item Other Symptoms (OSE) subscales from the Arthritis SE scale (ASE) were collected from the ALED participants at baseline and at 20 week follow-up; again this study uses the baseline data. The ASE scale was developed by Lorig and colleagues to measure a respondents' SE for arthritis self-management behaviors (e.g., decreasing pain, keeping pain from interfering with normal activities, and dealing with the frustration of having arthritis) [22]. These two subscales are estimated to take approximately five minutes to complete. The 9-item Function subscale is the third subscale of the ASE but was not collected in ALED [21]. The items were scored with a 10-point response pattern, with one representing “very uncertain” and 10 “very certain.” Lorig et al. found the 5-item PSE and the 6-item OSE subscales both sensitive-to-change when evaluating the Arthritis Self-Management course using the ASE [22]. The baseline scores in the ALED study are PSE 6.63 (SD 2.06) and OSE 6.94 (2.14).

Table 1 displays the items from the RASE and ASE utilized in this study.

### 2.3. Analysis

The goal of this series of analyses is to obtain IRT-based item parameters for both the ASE and RASE. Our original intention was to perform a unidimensional IRT analysis of both scales. Although published literature suggests that each scale exhibits multidimensionality, it is often the case that different approaches will yield different results [20, 22]. Even if the scales are found to be multidimensional, there are a number of strategies available to handle such a scale. We therefore performed the analyses with an eye towards identifying unidimensional scales, while being mindful of the potential for multiple dimensions. Exploratory and confirmatory factor analyses (EFA and CFA) were used to assess the extent to which a one-dimensional model could adequately explain the observed item responses. EFAs were conducted in CEFA using ordinary least squares (OLS) estimation, polychoric correlations, and oblique quartimax rotations (where necessary) [23]. In the EFA we focused on the scree plots, and if there was evidence of more than one factor, then we focused on the resulting factor loading matrix. The CFAs were conducted in LISREL, again with polychoric correlations, but this time using diagonally weighted least squares (DWLSs) estimation to provide correct fit indices (see Wirth and Edwards, 2007, for a more detailed description) [24, 25]. There are a number of fit indices available when conducting structural equation modeling-based CFA, but we have found a combination of the root mean square error of approximation (RMSEA), comparative fit index (CFI), and the root mean square error (RMSE) providing a nice balance of information regarding how well the model accounts for the observed data [26, 27]. RMSEA values less than 0.05 are viewed as indicating good model fit, values between 0.05 and 0.1 indicate moderate model fit, and values greater than 0.1 generally indicate poor model fit. CFI values greater than 0.9 indicate reasonable model fit with values over 0.95 indicating good model fit. RMSE values less than 0.1 indicate good model fit. We favor the RMSEA, CFI, and RMSE (in that order) as indicators of fit given the existing literature on model fit. 

Once a sufficiently unidimensional set of items had been identified, an IRT analysis was performed on each scale using the graded response model (GRM) as implemented in the Multilog software package [28, 29]. Following the IRT analysis we examine the estimated item parameters, standard error curve (SEC), and test information function (TIF) to better understand both how individual items are contributing to the scale and how the scale is functioning as a whole. Prior to any factor analytic or IRT analyses we collapsed any category which was chosen by less than 2% of the respondent. This led to no collapsing on the ASE (which was surprising, given that each item had 10 response categories) and minimal collapsing on the RASE.

This study was approved by the University of North Carolina Biomedical institutional review board and it was conducted with the understanding and the consent of the human subjects.

## 3. Results and Discussion

The analyses proceeded differently for the ASE and the RASE scales and in light of this we present the results from each in separate sections below.

### 3.1. ASE Results and Discussion

**Figure 1:**
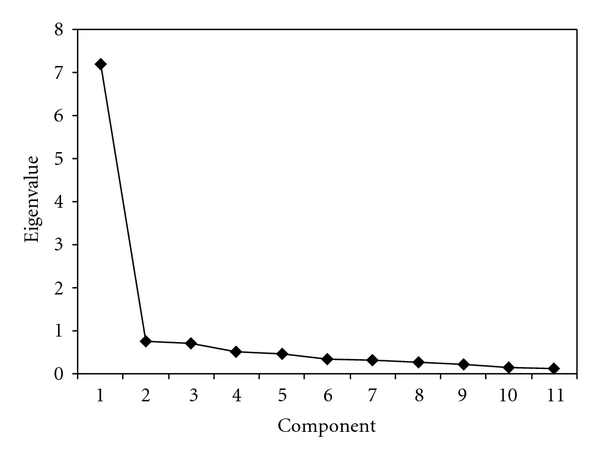
Scree plot for the 5-item Pain and 6-item Other Symptoms subscales from Arthritis Self-efficacy scale.

The initial validation study on the ASE found evidence for two and three factors. We focused on the items comprising what Lorig et al. titled the PSE and OSE subscales [22]. Although these were found to constitute two separate factors in the original study, our results suggest that they are adequately explained by one factor. The scree plot from these 11 items is shown in Figure 1. The scree plot suggests that there is one dominant factor. A one-factor model was fit in a CFA framework to assess model fit. The fit of the one factor model to the 11 items was poor (RMSEA = 0.14, CFI = 0.96, RMSE = 0.6), at least judging by the RMSEA, which is the fit index we tend to focus on. There was some evidence in this solution for two locally dependent item pairs (1 & 2 and 4 & 5). LISREL automatically calculates modification indices (MIs) for parameters that are constrained in a particular model. In theory, they are chi-square distributed with one degree of freedom and represent the expected improvement in model fit if a particular parameter was freely estimated. The covariances among the residuals are typically constrained to zero in CFA models. Large MI values for particular residual covariances suggest that, even after accounting for their shared relationship to the latent construct, items are more related to one another than the model predicts. We removed one item from each pair (1 & 5) and reran the model with the remaining nine items. This model seems to adequately explain the observed data (RMSEA = 0.06, CFI = 1.0, RMSE = 0.2). 

Before moving to an IRT analysis, we wanted to be sure that the two-factor model was not more appropriate for these data. We fit a basic two-factor model and then, when the same evidence for locally dependent pairs arose, we added correlated errors to accommodate that excess covariance. Although the two factor model with two correlated errors fits well (RMSEA = 0.06, CFI = 0.99, RMSR = 0.2), the correlation between the two factors was estimated at 0.95. A correlation of this magnitude strongly suggests that those two factors are, in fact, one factor. 

**Figure 2:**
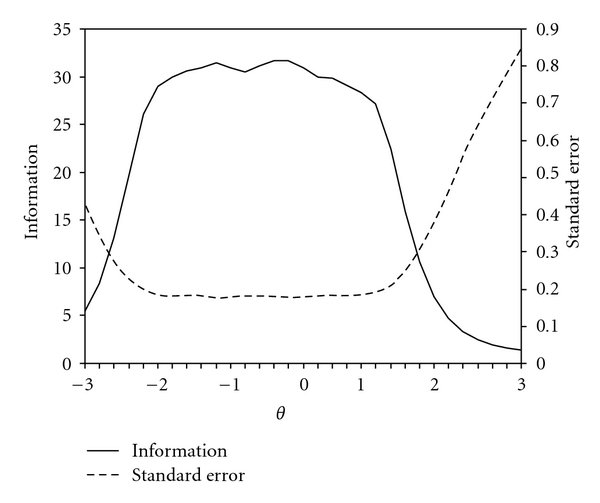
Information and standard error curves for a modified 9-item version of the the 5-item Pain and 6-item Other Symptoms subscales from Arthritis Self-efficacy scale.

Based on the strength of the factor analytic results we performed a unidimensional IRT analysis. In keeping with the results from the one-factor CFA, we omitted Items 1 and 5 from the IRT analysis. The parameter estimates from that analysis are given in Table 2. Although some of the slope parameters are high, subsequent analyses suggest that they are not inflated due to local dependence. The SEC and TIF for the modified 9-item version of the ASE are shown in Figure 2. As can be seen here the resulting scale provides highly reliable scores between −2.5 and 2 standard deviations. The precision quickly drops as scores increase above 2, as is noted by the increasing standard error curve and decreasing information curve. The marginal reliability for the nine-item scale was 0.95. 

The factor analytic results suggest that, despite published literature to the contrary, the PSE and OSE subscales from the ASE can be adequately accounted for by one underlying construct [22]. We identified two locally dependent pairs of items and dealt with this by removing two items. In addition to alleviating the local dependence, this has the added benefit of shortening the scale slightly.

### 3.2. RASE Results and Discussion

The EFA results showed not only one dominant eigenvalue (11.0), but also two other sizeable subsequent eigenvalues (2.9 & 2.1). A three-factor solution was estimated, but the resulting factors did not appear coherent from a substantive standpoint. One- and three-factor models were fit in a CFA framework to provide fit indices. The one-factor model did not fit particularly well (RMSEA = 0.09, CFI = 0.95, RMSR = 0.11), but a three-factor model with a few cross loadings provided an appreciably better fit (RMSEA = 0.5, CFI = 0.98, RMSR = 0.08).  Table 3 contains the factor loadings from this three-factor model. Despite the reasonable fit of this model, we found the lack of substantive coherence to be troubling.

The original validation study of the RASE suggested that it had eight factors and an additional three “orphan” items which did not load on any of those eight factors [20]. We attempted to replicate their final model in a CFA framework, but the estimator converged to an inadmissible solution. Although several attempts were made to modify this model, all resulting solutions were inadmissible. 

At this point, we went back to the items themselves and performed our own categorization process, where the number of factors and factor structure was determined based on a reading of the items. This led us to a nine-factor solution. We fit this model in a CFA framework and the model fit quite well (RMSEA = 0.03, CFI = 0.99, RMSR = 0.08). In an attempt to better understand the structure of this scale, we then fit a second-order factor model where a higher-order factor was underlying the nine lower order factors. While no direct comparisons between this and the base nine-factor CFA are possible (the models are unfortunately not nested), we note that the second-order model did account for these data reasonably well (RMSEA = 0.05, CFI = 0.98, RMSR = 0.1). 

These results suggest that although there may be one common construct underlying the responses to the items found on the RASE, it does so through nine subfactors. To the extent that there are different numbers of items representing each of these subfactors, the resulting summed score will be a weighted combination of them. In an effort to avoid this weighting and to see if it would be possible to perform a unidimensional IRT analysis on a subset of the 28-item RASE, we selected one item from each of the nine subfactors. When choosing items, we tried to balance statistical characteristics (choosing items with high factor loadings in earlier analyses) and content validity (insuring that the resulting collection of items had face validity). The fit of a one-dimensional model for these nine items was then assessed using CFA. This model fits the data well (RMSEA = 0.06, CFI = 0.99, RMSR = 0.06), which suggests that for this nine-item subset, unidimensionality is a plausible assumption. 

**Figure 3:**
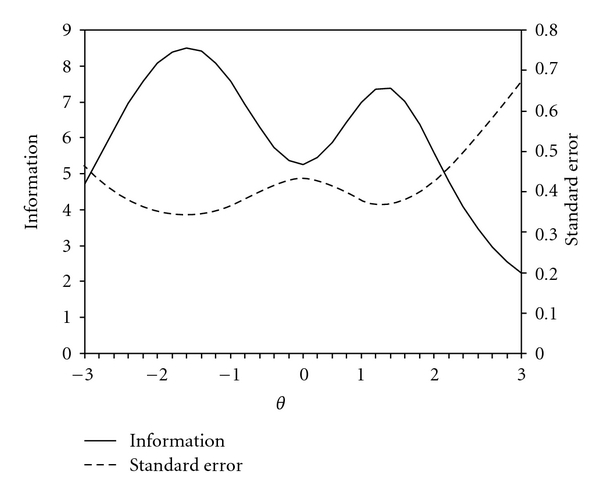
Information and standard error curves for a modified 9-item version of the Rheumatoid Arthritis Self-efficacy scale (RASE).

An IRT analysis was then conducted on those nine items. The resulting scale had a marginal reliability of 0.84 and with the exception of one item, all slopes were greater than one (item parameters are provided in Table 4). As indicated in Figure 3, the nine-item subset has a relatively uniform level of measurement precision (standard errors between 0.3 and 0.4) between −3 and +2 standard deviations. 

The factor analytic work for the RASE was substantially more complex than for the ASE. Neither the one-dimensional model we were hoping for nor the eight-dimensional model presented in the literature provided an adequate explanation of the RASE data. We went back to the item content created our own “bins” into which the items appeared to fall, which led us to a nine factor model. This model had good fit to the data and an additional higher-order model also had good fit. As previously mentioned, these two results suggest that while there may be nine subfactors, they are all related to some overarching latent factor. We proceeded by choosing one item from each subfactor to serve as the representative item for the subfactor on a shortened RASE.

### 3.3. Limitations

The two populations here are from the Southeastern US and both populations have similar demographics that are somewhat homogenous (i.e., primarily female, educated, and Caucasian). The retrospective recall reliance of these self-efficacy measures is a limitation especially for the RASE which has in its direction “even if you are not actually doing it at the moment” [20]. These scales are only analyzed cross-sectionally because the analyses proved to be much more complex determining the ability for each of these scales to detect change to be too in-depth for one manuscript. Cross population comparisons were not possible because we did not have data on both measures in one sample. We originally planned to equate these two arthritis SE scales but the wording variations were slight enough not to allow common-item equating procedures [30]. Although we were not successful, our results may be informative to future researchers who wanted to utilize common-item procedures on these scales.

## 4. Conclusion

We acknowledge that there are more complex solutions for a scale like the RASE. However, the alternative proposed here (the modified 9-item RASE) has the virtue of being shorter, representative of the construct of interest, and easy to implement with currently existing IRT software. In summary, these results show that, if necessary, unidimensional IRT could be used with a scale exhibiting the complex hierarchical structure of the RASE.

While the 9-item modified version of the two ASE subscales presented here is very effective at measuring much of the range of arthritis self-efficacy, it is not precise for individuals with very high levels (>2 standard deviations above the mean) of arthritis self-efficacy. The same holds for our modified 9-item version of the two RASE subscales. However, considering the very small number of individuals we would anticipate to have scores to be high (roughly 2.5%); this is not a serious weakness. When it would potentially become problematic is if either scale were being used to assess a treatment which was highly effective. In this case, either scale may exhibit a ceiling effect which could mask improvement beyond a certain level. Although any comparison between the scales must be made with caution, it does appear that the 9-item modified version of the two ASE subscales is able to provide more precise estimates than the modified 9-item RASE. This study is a first step towards increasing the precision of identifying those people with arthritis and low SE. This information may better inform SE-enhancing interventions [12].

## Figures and Tables

**Table 1:** Content of Rheumatoid Arthritis Self-efficacy scale (RASE) and Arthritis Self-efficacy scale (ASE) scales.

ASE subscales item number and content^a^:5-item Pain (items 1-5) and 6-items Other Symptoms (items 6-11)	
(1)	decrease your pain quite a bit?
(2)	continue most of your daily activities?
(3)	keep arthritis pain from interfering with your sleep?
(4)	make a small to moderate reduction in your arthritis pain by using methods other than taking extra medication?
(5)	make a large reduction in your arthritis pain by using methods other than taking extra medication?
(6)	control your fatigue?
(7)	do something to help yourself feel better if you are feeling blue?
(8)	regulate your activity so as to be active without aggravating your arthritis?
(9)	deal with the frustrations of arthritis?
(10)	As compared with other people with arthritis like yours, how certain are you that you can manage arthritis pain during your daily activities?
(11)	manage your arthritis symptoms so that you can do the things you enjoy doing?

RASE item number and content^b^	

(1)	use relaxation techniques to help with the pain.
(2)	think about something else to help with pain.
(3)	use my joints carefully (joint protection) to help with pain.
(4)	think positively to help with pain.
(5)	avoid doing things that cause pain.
(6)	wind down and relax before going to bed, to improve my sleep.
(7)	have a hot drink before bed, to improve my sleep.
(8)	use relaxation before bed, to improve my sleep.
(9)	pace myself and take my arthritis into account to help deal with tiredness.
(10)	accept fatigue as part of my arthritis.
(11)	use gadgets to help with mobility, household tasks, or personal care.
(12)	ask for help to deal with the difficulties of doing everyday tasks.
(13)	do exercises to deal with the difficulty of doing everyday tasks.
(14)	plan or prioritize my day to deal with difficulties of doing everyday tasks.
(15)	educate my family and friends about my arthritis to help with the strains that arthritis can make on relationships.
(16)	explain to friends and family when I do or do not need help.
(17)	discuss any problems with my partner or family.
(18)	make time for leisure activities, hobbies, or socializing.
(19)	save energy for leisure activities, hobbies, or socializing.
(20)	focus on the positive when I am feeling down.
(21)	use relaxation to deal with worries.
(22)	allocate time for relaxation.
(23)	use a relaxation tape or instructions to help me relax.
(24)	use regular exercise.
(25)	be aware of my limits in exercise.
(26)	manage my medication, knowing how and when to take it.
(27)	look out for and avoid side-effects of my medication.
(28)	seek help with persistent side effects.

^a^With the exception of item 10, all ASE items begin with “How certain are you that you can…”

^b^All RASE items begin with “I believe I could…”

**Table 2:** IRT parameters for the 9-item modified version of the 5-item Pain and 6-item Other Symptoms subscales from the Arthritis Self-efficacy scales (ASE).

Item	a	b1	b2	b3	b4	b5	b6	b7	b8	b9
1										
2	2.09	−2.43	−1.91	−1.61	−1.32	−0.83	−0.40	−0.13	0.38	0.88
3	1.90	−1.91	−1.46	−1.16	−0.82	−0.43	−0.01	0.32	0.86	1.36
4	2.33	−2.20	−1.80	−1.33	−1.00	−0.54	−0.24	0.05	0.66	1.19
5										
6	2.34	−1.93	−1.58	−1.22	−0.85	−0.36	0.01	0.30	0.69	1.21
7	2.81	−2.24	−1.77	−1.39	−1.29	−0.76	−0.45	−0.21	0.20	0.66
8	3.59	−2.02	−1.76	−1.37	−0.95	−0.48	−0.20	0.05	0.64	1.19
9	3.67	−2.13	−1.69	−1.27	−1.00	−0.58	−0.27	−0.01	0.45	0.97
10	5.10	−2.15	−1.71	−1.28	−1.09	−0.61	−0.33	−0.07	0.43	0.99
11	4.23	−2.10	−1.57	−1.23	−0.98	−0.54	−0.22	0.07	0.48	0.99

**Table 3:** Factor loadings from 3-factor Rheumatoid Arthritis Self-efficacy scale (RASE) model.

Item	Factor 1	Factor 2	Factor 3
1	—	0.75	—
2	—	0.7	—
3	—	0.75	—
4	—	0.75	—
5	—	0.64	—
6	—	0.57	—
7	—	0.44	—
8	—	0.73	—
9	—	0.46	0.39
10	—	—	0.54
11	—	—	0.62
12	—	—	0.68
13	0.46	0.33	—
14	—	0.41	0.47
15	—	—	0.77
16	—	—	0.65
17	0.65	—	—
18	0.81	—	—
19	0.48	0.34	—
20	0.52	0.31	—
21	0.43	0.4	—
22	0.83	—	—
23	—	0.67	—
24	0.62	—	—
25	0.72	—	—
26	0.75	—	—
27	0.74	—	—
28	0.72	—	—

**Table 4:** Item parameters for 9-item Rheumatoid arthritis self-efficacy scale (RASE) subset.

Item	a	b1	b2	b3	b4
3	1.59	−1.81	−0.97	1.35	
8	1.40	−2.99	−1.55	−0.64	1.91
10	0.70	−4.22	−2.05	−0.69	4.11
13	2.24	−2.47	−1.45	1.01	
14	2.23	−1.79	−1.03	1.18	
15	1.13	−3.57	−1.94	−0.93	2.14
19	1.94	−1.79	−1.06	1.39	
20	2.10	−1.98	−1.42	1.03	
26	1.42	−2.51	0.56		

## List of Abbreviations

SE: Self-efficacy
IRT: Item response theory
RCT: Randomized controlled trials
RASE: Rheumatoid arthritis self-efficacy scale
PACE: People with Arthritis Can Exercise
ASE: Arthritis self-efficacy scale
ALED: Active Living Every Day
EFA and CFA: Exploratory and confirmatory factor analysis
CHAMPS: Community Healthy Activities Model Program for Seniors
PSE: Pain subscale from the Arthritis self-efficacy scale
OSE: Other Symptoms subscale from the Arthritis self-efficacy scale
CEFA: Comprehensive exploratory factor analysis
OLS: Ordinary least squares
LISREL: Linear structural relations
DWLS: Diagonally weighted least squares
RMSEA: Root mean square error of approximation
CFI: Comparative fit index
RMSE: Root mean square error
GRM: Graded response model
SEC: Standard error curve
TIF: Test information function.
